# Smart Compression Therapy Devices for Treatment of Venous Leg Ulcers: A Review

**DOI:** 10.1002/adhm.202200710

**Published:** 2022-07-01

**Authors:** Gustavo Coelho Rezende, Brendan O'Flynn, Conor O'Mahony

**Affiliations:** ^1^ Tyndall National Institute University College Cork Cork T12 R5CP Ireland; ^2^ SWaT Research Network Member RCSI University of Medicine and Health Sciences Dublin D02 YN77 Ireland

**Keywords:** microsystems technology, smart compression therapy devices, venous leg ulcers, woundcare

## Abstract

Venous leg ulcers can have significant social and economic impacts, and are generally treated by applying compression to the lower limb, which aids in promoting blood return to the heart. Compression therapies commonly involve the use of passive bandages that suffer from issues associated with incorrect application, and although automated solutions have begun to appear; these are often bulky and hinder mobility. Emerging microtechnologies and new materials enable the development of “smart” compression therapy devices, which are defined as systems that use miniaturized and lightweight actuators and electronics to control the applied pressure. This paper reviews the state of the art in smart compression therapy research. A total of seventeen different devices has been identified, categorized as using one of three actuation mechanisms: pneumatic compression, motor‐driven mechanisms, and smart materials (including shape memory alloys, shape memory polymers, and electroactive polymers). The field is still in its relative infancy and further refinements are required to create mass manufacturable compression dressing systems that meet medical, ergonomic, and economic standards. The use of miniaturized actuators has immense potential for the development of smart compression dressings, which will ultimately lead to higher compliance, increased patient comfort, enhanced mobility, and better treatment outcomes.

## Graphical abstract

This paper reviews the emerging microtechnologies and new materials that enable the development of “smart” compression therapy devices, which are defined as those systems that use miniaturized and lightweight actuators and electronics to control the pressure applied to a limb. The technology has immense potential for the treatment of venous leg ulcers.

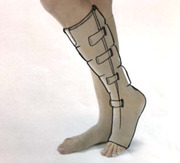

## 1 Introduction

Venous leg ulcers (VLU) are open sores that usually occur between the ankle and knee. Chronic leg ulcers are defined as those that persist for more than 6 weeks and show no tendency to heal after 3 or more months; in fact, many leg ulcers never heal.^[^ ^1^, ^2^ ^]^ The main cause of VLUs is high pressure in the veins of the leg, created by poor blood circulation arising from conditions such as defective venous valves, vascular malformations, and blocked veins (e.g., from blood clots).^[^ ^3^, ^4^, ^5^ ^]^ The resulting high pressure damages small blood vessels, which become fragile. As a consequence, even minor skin injury, such as that from an impact or scratch, can cause the skin to break easily and form an ulcer.^[^ ^6^, ^7^ ^]^

Severe non‐healing wounds, including VLUs, can have a large impact on economic resources. In developed countries, non‐healing wounds have a prevalence rate of 1% to 2% of the general population.^[^ ^8^, ^9^ ^]^ Guest et al.^[^ ^8^ ^]^ evaluated the level of healthcare resources used to treat and manage wounds in the UK, and found that management of wounds and comorbidities in 2021/2013 required 18.6 million practice nurse visits and 7.7 million general practitioner (GP) visits. Moreover, the annual cost to the National Health Service (NHS) of managing wounds and comorbidities was £5.3 billion at that time.^[^ ^8^ ^]^ Factors that can aggravate this scenario include an aging population and the presence of risk factors for atherosclerotic occlusions such as smoking, obesity, and diabetes.^[^ ^1^ ^]^

A common treatment for VLU is compression therapy, which uses external compression of the leg to improve circulation by promoting blood return to the heart,^[^ ^10^, ^11^ ^]^ and has been shown to significantly improve the quality of life in patients with chronic venous disease at all its stages.^[^ ^12^ ^]^ Application of the correct pressure is crucial for effective compression therapy,^[^ ^13^ ^]^ since low pressure delays treatment and high pressure can cause extremely low circulation, leading to pain or damage to the limb.^[^ ^14^ ^]^ The pressure levels necessary to treat VLU are typically up to 60 mmHg, and vary according to patient conditions such as arterial insufficiency, neuropathy, or cardiac failure.^[^ ^14^, ^15^, ^16^, ^17^ ^]^ In order to facilitate the blood to return to the heart, the pressure applied on the leg may be tapered, with higher pressure at the ankle and decreasing toward the knee.^[^ ^18^ ^]^ Applying the correct compression level and ensuring patient compliance are challenges commonly faced by health practitioners and therefore a trained healthcare professional must usually be present to apply the compression therapy.^[^ ^13^, ^19^ ^]^ Compression therapy for VLU treatment currently includes the use of bandages, compression stockings, and pneumatic compression devices (PCD).^[^ ^20^, ^21^ ^]^ **Table** **1** compares the advantages and disadvantages of those three types of treatment.

**Table 1:** Common commercial compression therapy equipment

Compression therapy	Structure	Advantages	Disadvantages
Bandage^[^ ^22^, ^38^, ^39^ ^]^	Short‐ or long‐stretch materials, wrapped around the affected limb using multiple layers and/or materials.	Size and pressure can be adjusted according to the patient's limb size and shape; lightweight and unobtrusive; user can move around.^[^ ^20^ ^]^	Success of application (i.e., precise pressure level) relies on training of medical staff and manual skills; may suffer pressure loss in the first hours due to wear;^[^ ^17^ ^]^ pressure may vary significantly depending on the patient movements.^[^ ^40^ ^]^
Stocking^[^ ^41^ ^]^	Hosiery‐type garments, including knee‐high socks, tights, and pantyhose.	Patients can generally don and doff stocking without assistance (with the exception of patients with mobility issues); stockings maintain pressure for longer time compared to bandage; are less dependent on manual skill compared to bandages; user can move around.	Skin allergies may present a problem; size must be prescribed according to user condition/size; not fully adaptable to limb shape.
Pneumatic compression device^[^ ^28^, ^42^ ^]^	Active devices, using electrical power and pneumatic pumps to selectively inflate chambers arranged in a boot‐like structure.	Automated compression; requires significantly less wear time; can be used in ulcers that do not respond well to conventional therapy; compression time and cycles can be adjusted to individual needs.	Venous stasis can occur; may reduce limb mobility; usually requires external inflation source connected by tubing; may be bulky and noisy; may prevent mobility.

Compression bandaging involves the spiral wrapping of up to four layers of bandage over the affected limb in order to provide a graduated pressure profile. Although bandages are lightweight and low cost, the success of this technique depends strongly on the individual skill of the nurse, which makes it difficult to reliably achieve the desired compression values.^[^ ^13^, ^22^ ^]^ Factors such as bandage elasticity and stiffness indices, which quantify the difference in sub‐bandage pressure between standing and resting states,^[^ ^23^ ^]^ are key characteristics in the treatment of VLUs. In general, compression bandages can be divided into long stretch bandages (LSBs) and short stretch bandages (SSBs); LSBs may be extended by over 100% compared to their original length, while SSBs are less extensible. The relatively elastic LSBs generate moderate pressures that are similar during the resting and standing states, while SSBs provide low resting pressures that significantly increase in the standing position due to muscle contraction. In addition, the stiffness of the SSB material generates high sub‐bandage pressures during exercise, which provides a massaging effect during walking.^[^ ^21^ ^]^

However, bandages may suffer from loss of elasticity with time and washing, and incorrect application can result in treatment delay, limb distortion, and bandage slippage.^[^ ^14^, ^21^ ^]^ Stockings—including knee‐length socks and tights—provide the user with a higher degree of freedom to self‐apply the treatment, and rely less on manual skill for application. However, the pressure level depends on the user's limb shape/size.^[^ ^24^, ^25^ ^]^ Both bandages and stockings are manufactured using a range of fibers and yarns, which are used to create elastic fabrics using knitting techniques, most commonly weft and warp knitting. Both the type of yarn and knitting construction may be varied in order to fabricate a wide range of textiles that are used to provide elasticity, stiffness, and pressure levels tailored to the clinical requirements of each individual patient.^[^ ^26^, ^27^ ^]^

In order to ensure precise pressure control and improve compliance, a device that can monitor and adjust the compression is highly desirable. PCDs offer this possibility^[^ ^28^, ^29^, ^30^ ^]^ by using a pumping unit to inflate one or more bladders mounted on a garment in order to compress the leg. A wide range of PCDs exist and can be classified into three types: 1) fixed PCDs with no mobility, where the pumping unit is a large stationary unit and the patient cannot move around while wearing the garment;^[^ ^31^ ^]^ 2) semi‐portable PCDs with extended mobility, where the pumping unit is small and can be carried around;^[^ ^32^ ^]^ 3) portable PCDs, where the pumping unit is integrated into the garment and allows the patient to move freely while the device is operating;^[^ ^33^, ^34^, ^35^, ^36^ ^]^ Some PCDs use massage cycles to improve circulation, which might accelerate VLU treatment. As an emerging alternative to PCDs, Elastimed^[^ ^37^ ^]^ developed a portable garment using electroactive polymers (EAPs) as actuators.

The ultimate solution in compression therapy would maintain optimum pressure levels, ensure compliance and enable the user to live a near‐normal life whilst wearing the device. This intelligent system would continuously monitor and adjust the pressure in response to the user's activity levels, and furthermore would wirelessly transmit the relevant compression data to healthcare professionals. Forming part of the Internet of Medical Things (IoMT),^[^ ^43^, ^44^ ^]^ the “smart compression therapy (SCT) device,” would remotely monitor treatment progress and wound status in home‐care settings.^[^ ^45^ ^]^ Eventually, embedded sensors could also be incorporated for monitoring wound parameters such as exudation rate and infection control.^[^ ^46^ ^]^ Furthermore, SCT devices could help to increase adherence to treatment programs, as a large proportion of patients are non‐compliant with compression therapy.^[^ ^19^, ^47^ ^]^ An easy‐to‐apply device with adjustable pressure levels could improve compliance by reducing some of the adverse events associated with bandages and wraps, most notably skin irritation and the feelings of discomfort or pain commonly reported by patients. These are often caused by incorrectly applied devices such as wraps that are initially applied too tightly, or that may be worn for too long.^[^ ^48^ ^]^ It has also been emphasized that patient compliance may be increased by engagement and education.^[^ ^21^ ^]^ Although conclusive data on its effectiveness is still scarce, the use of interactive tools such as gamification and loyalty‐style incentives is already being investigated in many healthcare settings,^[^ ^49^, ^50^ ^]^ and it is reasonable to assume that SCT devices could use smart sensors and a wireless interface to engage with the patient in a similar manner.

Therefore, some design requirements for such an SCT device include, but are not limited to 1) the ability to provide compression within the desired pressure range; 2) low energy consumption; 3) rapid pressure adjustment; 4) wireless connectivity; 5) continuous monitoring of pressure levels; 6) a lightweight and comfortable form factor. The requirements for portability and adjustability mean that a miniaturized and lightweight actuator will be at the core of these SCT devices. The actuator will directly or indirectly provide the forces required to compress the limb, and could take the form of a pump, motor, smart material, or other miniature and controllable device.

This paper identifies recent developments in portable compression therapy devices that use miniaturized actuators for automatic compression. Some of the innovative devices presented may be intended to treat other infirmities besides VLUs, since compression therapy can be used for multiple purposes such as sensory processing disease, orthostatic hypertension, diabetes, lymphoedema, burns, and sports recovery.^[^ ^51^, ^52^, ^53^, ^54^ ^]^ However, all devices mentioned in this paper could, in principle, be adapted for VLU treatment. The main components and characteristics of the treatment devices are described, including actuators, sensors, communication systems, construction materials, and performance. A discussion is provided on the advantages and disadvantages of each of the actuation mechanisms, followed by an outlook for future development requirements. It is clear that this remains an emerging field, but one that has significant potential for high social and economic impact if current rates of progress toward new SCT devices can be maintained.

## 2 State of the Art: SCT Devices

This section reviews the emerging devices and systems that use miniaturized actuators to provide compression. A total of seventeen SCT devices have been identified, which may be categorized by operating principle into one of three types: pneumatic compression, motor‐driven mechanisms, and smart materials (including shape memory alloys (SMAs), shape memory polymers (SMPs), and EAPs).

### 2.1 Pneumatic Compression

**Figure 1:**
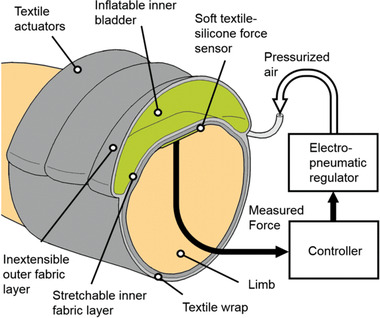
Compression device using a bladder to simulate a massage effect. Reproduced with permission.^[^ ^55^ ^]^ Copyright 2018, IEEE.

Several articles describe pneumatic compression mechanisms that are based on the use of a pump to increase the air pressure inside a bladder that is wrapped around the limb. Using this concept, Payne et al.^[^ ^55^ ^]^ developed a device to simulate a massage effect, **Figure** **1**. Intended to be placed around a limb and secured with Velcro, two versions of the device were tested: a first version with single actuator‐sensor module; and a second version with three actuator‐sensor modules to facilitate the massage effect. Each actuator‐sensor module was comprised of two discrete layers, one for sensing and another for actuation. The sensing layer was comprised of a soft force sensor sewn into the elastomeric wrap material. The sensor used a capacitive transduction mechanism.^[^ ^56^, ^57^ ^]^

The actuator comprised an SMC Pneumatics (CA, USA) pressure regulator, compatible with pressures up to 0.9 MPa, interfaced with a sleeve fabricated from two textile layers sewn together. The sleeve housed an inflatable bladder. The inner sleeve layer was sewn with an elasticated warp‐knitted raschel polyamide‐elastane textile, and the outer layer was a non‐stretchable Kevlar‐reinforced sailcloth. This arrangement of non‐stretchable outer and extensible inner layers directed the force inward toward the muscle, and also provided recoil to assist with deflation of the bladder. The bladder itself was made of a thermoplastic elastomer using a heat press. An airtight seal for the bladder is achieved by using a Loctite Vinyl fabrics adhesive. A National Instruments controller was used to enable closed‐loop control of the inner pressure of the bladder. The device could regulate sinusoidal force profiles with amplitudes up to 60 N with 0.7 N resolution of the desired force. Force‐controlled pressure gradients were generated using sequential actuation, mimicking a massage. Peak pressures of 10 kPa (75 mmHg) were observed from measurements of a pressure mat.

**Figure 2:**
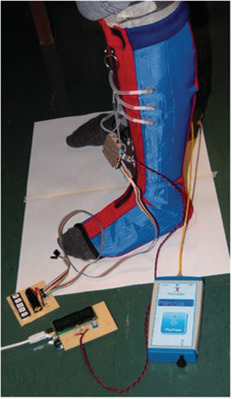
Compression garment using bladders made of polyester fabric coated with polyurethane. Reproduced with permission.^[^ ^59^ ^]^ Copyright 2018, Sage Publishing.

Micropump technologies could be used as a pathway to further miniaturize this bladder‐based approach to compression therapy. Modern micropumps are used for a wide range of biomedical applications, and are lightweight, low power, low cost, and offer good flowrate and pressure ranges.^[^ ^58^ ^]^ Hakala et al.^[^ ^59^, ^60^, ^61^ ^]^ developed a compression garment using bladders and micropumps as actuation mechanisms, **Figure** **2**. The main objective of the study was to observe graduated compression and its control, and to improve conventional PCDs by using smaller and lighter components that did not hinder motion or require the patient to remain still during treatment. The prototype had three pairs of bladders fabricated using impermeable polyester fabric coated with polyurethane, where a lighter coated fabric (170 g m^−2^) was used for the inner layer, and a heavier coated fabric (270 g m^−2^) for the outer surface. The fabric tenacities were isotropic in the weft and weave directions. A zipper was used in front of the device to ease dressing and undressing. The micropump provided a maximum air pressure of 400 mmHg and worked with a small valve to control the air flow. Flexible tubing connected the micropump to the plastic inlets of the air bladders, which were seamed together by high frequency welding. The total weight of the prototype system was 326 g and Bluetooth communication was established between the prototype and an e‐reader. A manometer read the pressure inside the air bladders and a closed‐loop algorithm was used to automatically control the pressure inside the bladder. A linear relation between air pressure inside the bladder and pressure on the skin was found. A commercial understocking with antibacterial properties was used between the leg and prototype to protect the fragile skin against friction. Another advantage was that the actuator was removable, if maintenance was required. The device was tested in a volunteer and in a leg model. In the leg model tests, pressure levels from 50 to 80 mmHg were reached in seconds. This device provided dynamic compression and had small holes in the warp knit structure for ventilation.

### 2.2 Motor‐Driven Mechanisms

**Figure 3:**
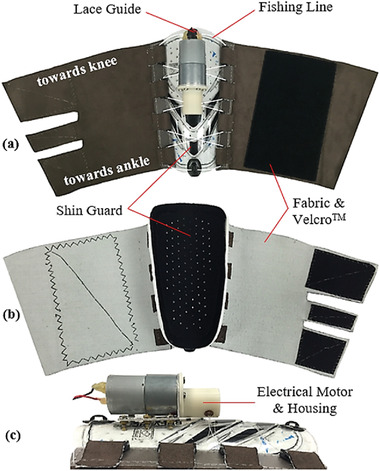
Compression device from Rahimi et al. using motor and shin guard. Reproduced with permission.^[^ ^62^ ^]^ Copyright 2017, Elsevier Ltd.

Some miniaturized motors are capable of providing enough torque to tighten a strap or line which in turn constricts the limb. Rahimi et al.^[^ ^62^ ^]^ developed a garment that applied pressure to the lower extremities using a 12 V DC minimotor (Pololu, USA) for actuation, and flexible force sensing resistors for pressure monitoring. A key goal was to sustain constant tension in the garment, thereby creating graduated pressure up the leg that decreased from ankle to calf, and that ensured blood movement toward the upper body. The device used a lace, made from 4‐ply fishing line, attached to the minimotor to tighten or loosen a piece of fabric. The motor was fixed on a soccer shin guard using an aluminum l‐bracket, **Figure** **3**. The spool and housing were 3D‐printed from ABS plastic and attached to the motor shaft. The garment used a non‐stretchable fabric; however, it was flexible enough to allow movements. The device could deliver sustained or intermittent pressure modes, was battery operated, and could be used during stasis or ambulation. Velcro secured the fabric on the limb, so that various leg sizes could be accommodated. The overall weight was 280 g. The pressurized state was reached in 2 s or less, with approximate maximal compression values of 160 and 180 mmHg over the mid and lower calf, respectively.

**Figure 4:**
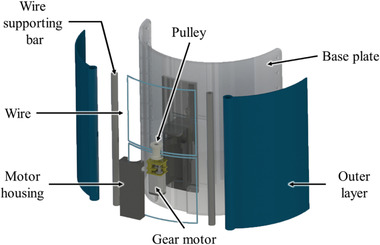
Compression device using a minimotor. Reprinted with permission.^[^ ^63^ ^]^ Copyright 2019, IOP Publishing.

Yang et al.^[^ ^63^ ^]^ also developed a compression device using a minimotor (Pololu, USA) as a wire actuation system, a soft pressure sensor, a feedback controller, and a two‐layer fabric structure as shown in **Figure** **4**. The garment was intended to provide variable compression levels in a lightweight and compact form factor. The outer layer housed a wire actuation system that generated a compressive force by pulling on both ends of a wire. Between the outer and inner layers, a soft pressure sensor, made of fabric to improve user comfort,^[^ ^64^ ^]^ was integrated. This sensor measured capacitance between two electrodes made of conductive fabric. The device was worn like a stocking and a spun‐silk fabric was used as an inner layer as it was soft and elastic when in direct contact with the skin. The outer layer used a single‐layer mesh fabric to provide stiffness in the direction in which the wire was pulled. As indicated in Figure 4, two bars were attached at the two ends of the outer layer in order to evenly distribute the tension exerted by the wires. The sleeve compression was controlled by the feedback mechanism that regulated the wire tension applied by the minimotor. A baseplate made of polyurethane was used to mount the minimotor housing. The sensing resolution of the device was 0.4 mmHg and the applied pressure varied from 19.5 to 87.3 mmHg.

### 2.3 Smart Materials

The electrical and mechanical properties of smart materials may be affected by external stimuli; thus, characteristics like shape and stress can be tuned by influences such as temperature, electrical or magnetic fields.^[^ ^65^, ^66^ ^]^ This behavior makes smart materials suitable candidates for use as actuators in compression therapy devices. The following subsection presents three types of smart materials used as actuators in compression devices: SMAs, SMPs, and EAPs.

#### 2.3.1 SMAs

SMAs are metallic alloys that present the shape memory effect. In other words, after being subjected to an initial shape setting or memorization step, they then can reversibly change form when a certain temperature is applied to the material (often using Joule heating).^[^ ^67^ ^]^ This is because such materials exist in different crystalline states at different temperatures—an austenite phase at high temperatures, and martensite phase at lower temperatures. After a programming or shape setting step is performed by subjecting the material to a high temperature anneal, the shape that the alloy was placed in during this process is retained even as it cools to the martensite phase. In this low‐temperature state, the structure can be plastically deformed into any other desired shape, but will recover its initial memorized shape when heated to a transition temperature between the martensite and austenite phases. Because of this ability to be programmed and to reversibly change shape, SMAs can be used as actuators and can also enable mechanical simplicity, reduced size, and biocompatibility—three important characteristics for integration into wearable medical devices.^[^ ^68^, ^69^, ^70^ ^]^

The most widely used SMA material is a nickel‐titanium (NiTi) alloy, also known as Nitinol. NiTi is used in all but one of the structures presented here, due to its excellent electrical and mechanical properties, corrosion resistance, long fatigue life, and biocompatibility. It is commonly available in wire form, with transition temperatures in the range of −100 to +100 °C, and the ability to recover plastic strains of ≈6–8%. When used as an actuator, NiTi is often shaped set at temperatures of 400–500 °C into a coil or spring‐like form, because of the ability of the coil to generate significantly larger displacements than the wire equivalent—several multiples of its initial contracted length. The forces exerted during the thermal shape recovery phase can be related to the geometrical characteristics of the coil and the metallurgical properties of the alloy material.^[^ ^71^, ^72^, ^73^, ^74^ ^]^

**Figure 5:**
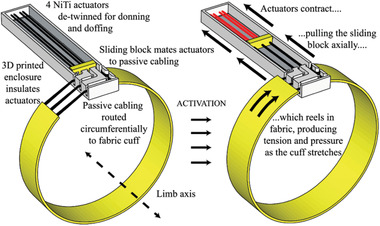
SMA coil used to drive compression in tourniquet made of breathable fabric. Reproduced with permission.^[^ ^72^ ^]^ Copyright 2015, IEEE.

Holschuh et al.^[^ ^72^ ^]^ presented a prototype tourniquet using NiTi coil actuators (**Figure** **5**), where the pressure was controlled using voltage regulation. The prototype had three main subcomponents: a) an arrangement of four NiTi coil actuators aligned in parallel; b) a band of 2.54 cm wide breathable fabric to form a tourniquet around the limb; and c) a 3D printed structure to isolate the actuator wires from the tourniquet system and to provide a sliding block for the actuator wires. These NiTi coils were formed from 305 µm diameter wire and shape set by annealing at 450 °C for 10 min, and a thorough model of the thermal and mechanical characteristics of the structure was also presented. In turn, this actuator tightened a series of 200 µm diameter stainless steel cables that formed the core of the tourniquet system. This architecture provided electrical and thermal insulation, compact and stable voltage‐based actuation, and an optimized fabric cuff for the wearer. Force and pressure tests were performed around a rigid cylinder: forces up to 24.75 N were measured at 5 V (4.55 W) actuation energy, and the pressure reached up to 6.05 kPa (45 mmHg) when the tourniquet was activated, an increase of 75% compared to the deactivated cuff. Pressure values stabilized for voltages above 6 V.

**Figure 6:**
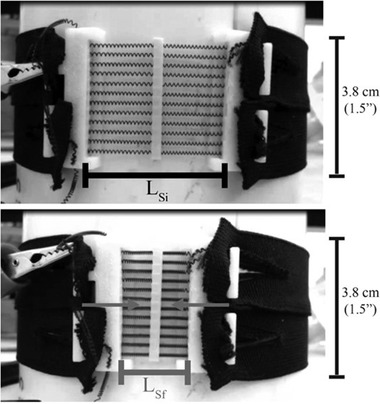
NiTi SMA coil actuators used in cuff‐like compression garment. Reproduced with permission.^[^ ^75^ ^]^ Copyright 2016, Aerospace Medical Association.

In a similar work, Holschuh et al.^[^ ^75^ ^]^ developed a cuff‐like compression garment (1.5" width) also using NiTi SMA coil actuators and a 3D printed modular cartridge to house the actuators, **Figure** **6**. The device delivered dynamic compression compatible with mechanical counter‐pressure suits for space exploration activities. The actuation cartridge used one SMA spring laced 12 times between its end caps, and one central spacer that was also 3D printed. This resulted in an equivalent actuator with 12 parallel springs to generate a better compression, and the design ensured structural stability and electrical conductivity. The cartridge was fabricated with a multi‐plastic method to enable greater thermal stability, and paired with a passive fabric to form a sleeve‐like garment. Jumbo and generic spandex fabrics were used to form the sleeve, although the generic fabric exhibited non‐linear stress–strain behavior that resulted in significant disagreement between modeled and experimental results. A disadvantage caused by the cartridge was that it created discontinuities in pressure. A power input of 27 W (30 V, 0.9 A) was required for 9 s to achieve the pressure targets, and the maximum counter‐pressure measured was 34.3 kPa (257 mmHg).

**Figure 7:**
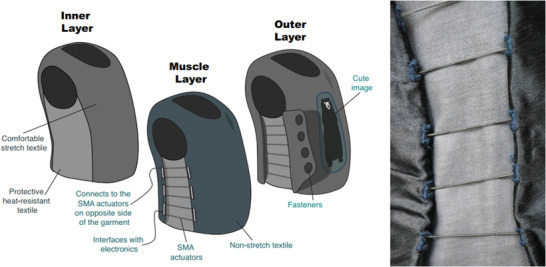
Children's deep pressure vest using SMA spring actuators. Reproduced with permission.^[^ ^76^ ^]^ Copyright 2016, ACM. This device is similar to another from the same team.^[^ ^53^ ^]^

Duvall et al.^[^ ^76^ ^]^ developed a children's deep pressure vest using SMAs made from 0.012″ Nitinol wire (shape set at 450 °C) as actuators, which is illustrated in **Figure** **7**. The same principle could also be adapted for the treatment of VLUs. This device was intended to treat sensory processing disorders, and used Bluetooth Low Energy communication to close a relay after which a current of 0.25 A ran through the wires when a 22 V battery was used. A 3D printed structure was used to house the electronics. The garment design had three main layers: 1) that closest to the user, made from heat‐resistant textile and Lycra to ensure low friction and thermal insulation from the SMA heat; 2) an actuation layer, housing the SMA wires and made from a non‐stretch textile; 3) an outside layer to protect the user from heat and enhance aesthetic aspects.

A similar device was introduced by the same team.^[^ ^53^ ^]^ That vest had 16 SMA spring actuators that constricted when heated using an applied electrical current. Each spring was 53.3 cm long when extended, had a diameter of 1.25 mm, and a spring index equal to 3. A single SMA spring was laced eight times from bottom to top of the garment, spaced at 1.9 cm vertically. This architecture was implemented at both sides of the vest, creating two actuation regions with an equivalent total of sixteen actuators. The total weight of the vest ranged from 0.4 to 0.6 kg, and a zipper on the front allowed easy don/doff. Similar to the previous device, the inner layer insulated the wearer from the heat generated by the SMA springs, whilst having low friction. Those target characteristics were met using a combination of Lycra textile, sewn with aramid and neoprene. In the experiments, voltages of 10 V to 20 V were applied per side (current ranging from 0.566 to 1.096 A and power from 5.81 to 21.92 W). As power increased, so did the average pressure, reaching up to 37.6 mmHg at 43.8 W.

**Figure 8:**
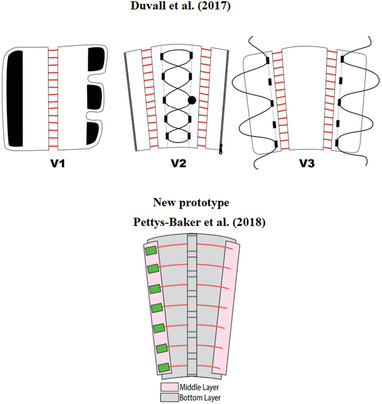
Garments for leg compression using shape memory alloy materials. Top: Reproduced with permission.^[^ ^77^ ^]^ Copyright 2017, ASME. Bottom: Reproduced with permission.^[^ ^78^ ^]^ Copyright 2018, ASME.

Duvall et al.^[^ ^77^ ^]^ also developed three prototype devices focusing on improving venous return for orthostatic intolerance. Each prototype had independent thigh and calf sections activated by SMA when heated. The garments were mostly constructed with rip stop nylon and cotton/aramid blends. The three versions are displayed in **Figure** **8**a and the maximum average pressures were 30, 17, and 17 mmHg respectively.

In a similar direction, Pettys‐Baker et al.^[^ ^78^ ^]^ designed and tested a calf and thigh garment capable of applying dynamic compression using SMA coils as actuators, Figure 8b. Comparing it with the original design of Duvall et al.,^[^ ^77^ ^]^ one main difference was the implementation of a tension switch, which provided automatic on/off compression. Second, the SMA coils were independently attached in horizontal rows. Moreover, the Pettys‐Baker device had compression generated by SMA actuators constricting around the leg, while in the previous versions presented by the Duvall team (Figure 8a), the actuators were used to tighten a passive fabric around the leg. The Pettys‐Baker et al.^[^ ^78^ ^]^ device used an inner layer to protect the leg from heat and distributed pressure, and Teflon sheets were employed for this purpose. A fiberglass ribbon tape was sewn vertically with several open channels to keep the actuators in place. The outer layer protected the actuator from outside forces and the user from the heat generated. The SMA was a 0.012″ diameter NiTi alloy wire coiled into a spring with a 0.048″ outer diameter and shape set at 400 °C for 10 min, and the temperature required to recover its original shape was 70 °C. No pressure measurements were reported.

Using Flexinol wire, Granberry et al.^[^ ^79^ ^]^ developed an SMA‐based knit compression stocking for orthostatic hypotension treatment of the lower leg. The device was designed to apply gradient compression from ankle to knee, and to be capable of generating pulsating sequential pressure. The stocking was activated by heat at temperatures of 70 °C. Flexinol NiTi wire was chosen because it provided consistent mechanical performance and is readily available at a reasonable cost.

**Figure 9:**
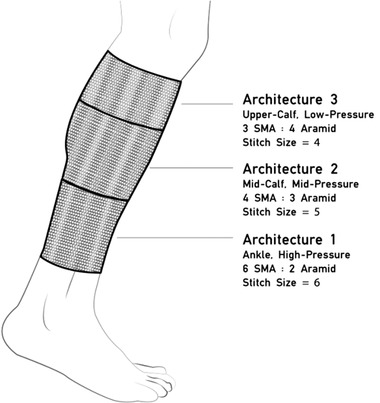
Knit compression stocking with varying proportions of SMA and base textile for gradient pressure application. Reproduced with permission.^[^ ^79^ ^]^ Copyright 2017, ACM.

The prototype was fabricated with a Passap E6000 double‐bed electronic knitting machine using a combination of 8 mil Flexinol NiTi wire and Kevlar aramid fiber yarn (Tex60). With a weft knit, the garment was constructed in three panels, each using a different stitch size and a different ratio of active NiTi wires to passive aramid yarns in order to generate a gradient pressure on the leg. A higher proportion of active yarn and larger stitch sizes generated larger forces and these panels were placed closer to the ankle, while fewer active yarns and smaller stitch sizes generated lower forces and were located higher on the leg. The panels were subsequently crocheted together to form a single garment, **Figure** **9**. This design provided a dynamic, untethered, and mobile stocking. Power of 40 W per panel was required, yielding a total power consumption of 120 W. A limitation of this design is the high activation temperature. However, it is possible to mitigate this by using SMAs with a lower activation temperature of ≈37 °C. No pressure data was reported for this proof‐of‐concept stocking.

**Figure 10:**
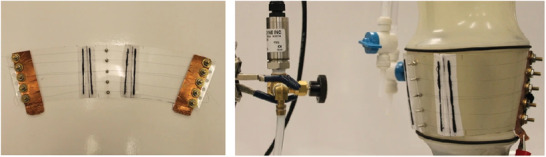
Compression device actuated by six SMAs and a plastic sheet. Reproduced under the terms of the CC‐BY 4.0 license.^[^ ^80^ ^]^ Copyright 2014, the Authors, published by Springer Nature.

Moein et al.^[^ ^80^ ^]^ developed a compression bandage intended for use in intermittent compression therapy, **Figure** **10**. Actuation was provided by six 102 µm diameter NiTi wires and a 0.6 mm thick polycarbonate sheet was used to apply the resultant pressure to a model of the calf. The wires were mechanically clamped to the sheet, copper tape was used to form electrical connections, and shoelaces were used to fasten the bandage with negligible strain. Experiments were performed with the same test setup proposed by Pourazadi et al.^[^ ^81^ ^]^ The total pressure exerted on the leg model was the sum of two components: 1) mechanical pressure, which resulted from wrapping the bandage around the limb; and 2) actuation pressure, caused by the heated SMA wires. SMA activation increased pressure by up to 40.8% (an increase of 9.06 mmHg).

**Figure 11:**
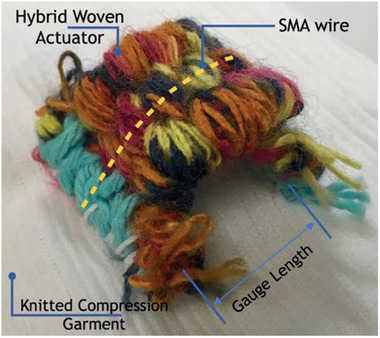
SMAs integrated inside a woven textile actuator. Reproduced with permission.^[^ ^82^ ^]^ Copyright 2017, CIMNE.

Kennedy et al.^[^ ^82^ ^]^ developed a textile‐based robotic compression garment for blood‐clot prevention, which was also capable of applying intermittent compression. The device was constructed using hybrid SMA‐textile actuators attached to commercial static‐compression sock fabrics using a cotton “cap” and 1 mm cotton thread. The textile itself used a plain weave and acrylic‐polyester braid yarn), **Figure** **11**. The authors used straight 60‐long, 0.5 mm diameter Ni‐Ti‐Cu SMA wires shape set at 500 °C for 1 h, which was part of the textile structure to enable good recovery and control of other features such as garment elastic displacement, safety, and temperature. To activate the textile actuator, a current of 2 A at 0.05 Hz frequency was applied. Heating and cooling times of 5 and 15 s were used, respectively. A thermocouple was used to measure the temperature of the woven actuator and compression garment. The design insulated the user from the SMA‐generated heat, resulting in a maximum temperature under the actuator of 36 °C and under the garment of 26 °C (when measured at a reference ambient temperature of 22 °C). The actuator caused a pressure increase ranging from 0.3 to 1.8 mmHg over the compression garment starting pressure.

#### 2.3.2 SMPs

Similar to SMAs, SMPs are defined as materials that are capable of “memorising” a permanent shape that is formed during initial fabrication, but that can be “programmed” using external mechanical force into a temporary state that may be maintained under appropriate conditions. Exposure to an external stimulus such as heat, electric fields, magnetic fields, or irradiation will then cause the structure to revert back to its permanent form.^[^ ^83^, ^84^, ^85^, ^86^, ^87^ ^]^ The most common SMP is thermally actuated shape memory polyurethane (SMPU), which is set into a permanent structure during initial processing at temperatures above the transition temperature of the material. After programming into its temporary shape, the original permanent shape may be restored once external forces are removed and the device is heated above the transition temperature.^[^ ^88^, ^89^ ^]^

Compared to SMAs, SMPs are low cost, easy to process and to integrate with textiles, can withstand higher elastic deformation, have lower actuation temperatures, and have physical properties similar to that of human tissue. However, SMP materials also have poorer mechanical performance, particularly with regard to recovery force (typical values for the stress generated during the recovery phase are 1–3 MPa for SMPs and 150–300 MPa for SMAs), and can be slow to recover because of their relatively low thermal conductivity.^[^ ^86^, ^87^, ^90^ ^]^

**Figure 12:**
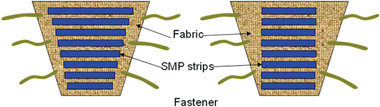
Shape memory polyurethane actuator in a compression bandage. Reproduced under the terms of the CC‐BY 4.0 license.^[^ ^91^ ^]^ Copyright 2012, the Authors,  published by Taylor & Francis.

Ahmad et al.^[^ ^91^ ^]^ investigated the use of SMPU actuators in compression bandages. Two designs were proposed as shown in **Figure** **12**, one featuring SMPU strips with fixed strain and different length and a second that used SMPU strips with fixed length but different strains. Using epoxy glue, these strips were attached to a knitted fabric that was intended to form part of the bandage underlay padding. The transition temperature (i.e., the temperature that, when exceeded, enables the material to return to its original shape) of the SMPU used was ≈50 °C. Heat was used as a stimulant, which in this case caused the SMPU strips to shrink and return to their original length. An advantage of this design was that the SMPU strips could be reused or recycled. The prototype was tested around a glass cylinder that was used to simulate a human leg model. The authors found that as the number of testing cycles increased, the applied pressure decreased. Pressure drop was also evaluated as a function of time, and in a test with 5 h duration, at 60  °C, the pressure fell from 33 to 4 mmHg (an 88% drop). In comparison, for the same 5 h at 30 °C, the pressure dropped only 3%.

**Figure 13:**
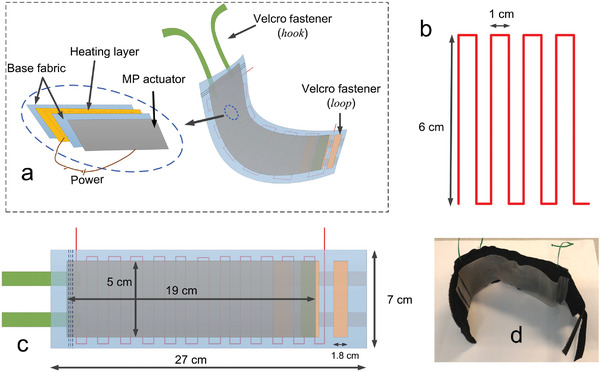
Compression device with dual function, heating, and compression. Reproduced with permission.^[^ ^92^ ^]^ Copyright 2016, Elsevier Ltd.

Kumar et al.^[^ ^92^ ^]^ investigated the potential of using memory polyurethane polymer (MPU) materials in compression devices with dual functions, that is, heating and compression. Their smart bandage used electrically resistive wires as heat sources for thermal therapy and MPU actuation for compression therapy; this device had both dynamic and static compression modes. A bandage prototype as shown in **Figure** **13** was developed that contained a base fabric (woven polyester yarn), a heating actuator unit (copper wire of 1.8 mm diameter), and a MPU actuator. The fabric layers secured the heating elements when stitched together. A potential disadvantage for MPU‐based bandages is low breathability. To solve that issue, the authors suggested that the MPU be converted into filaments and used within a fabric structure, which could have an improved porosity. Another disadvantage of MPUs is the slow activation speed—for example, commercial intermittent pneumatic compression massage cycles range from 30 s to 180 s, while MPU cycles take around 10 min. Results showed that temperature changes around 35 °C to 45 °C provided the greatest pressure differential, ranging from 0 to ≈40 mmHg. A theoretical model of the MPU performance was also presented.

**Figure 14:**
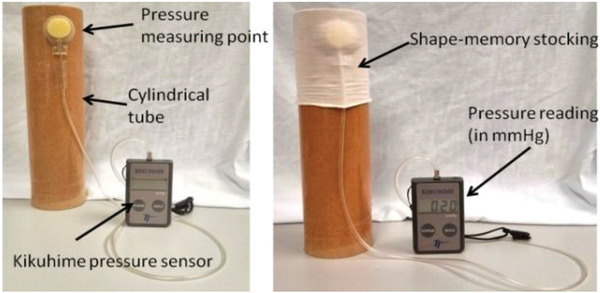
Compression stocking made from blend yarn of nylon and memory polymer (MP) filaments.^[^ ^93^, ^94^ ^]^ Reprinted with permission.^[^ ^93^ ^]^ Copyright 2016, Elsevier Ltd.

Also using SMP, Kumar et al.^[^ ^93^ ^]^ presented a stocking using a blended yarn of SMP (polyurethane) and nylon filaments, **Figure** **14**. The SMP developed had a low *T*_g_ of 30 °C and the activation range was chosen as between 30 and 50 °C. Melt spinning was used to develop the SMP filaments, and SMP (18.6 tex) and nylon (18.9 tex) yarns were combined to make the smart stocking using a circular knitting machine. The stocking was able to generate additional pressure up to 16.2 mmHg on top of a baseline pressure of 30 mmHg (i.e., a maximum total pressure of 46.2 mmHg).

Kumar et al. also reported a study^[^ ^94^ ^]^ in which the SMP filament was prepared via melt spinning with segmented polyurethane, and a V‐bed double jersey flat weft knitting machine was used to make a yarn blend of nylon (59.2%) and SMP (40.7%). The filament density was 18.9 tex for nylon and 13 tex for SMPU. In the experiments, the device was heated to 50 °C in a heating chamber, which increased the compression. It was noted that several other factors, primarily limb circumference and a number of bandage layers, also influenced the compression level. Pressure variations of up to 21.6 mmHg were observed as a function of temperature, number of layers, and applied strain.

**Figure 15:**
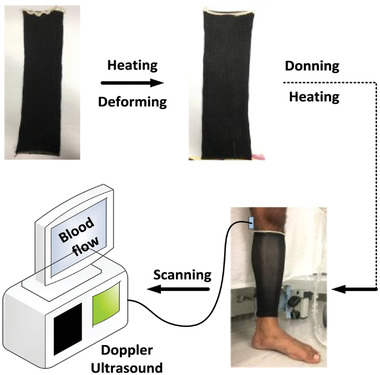
Smart stocking with SMP filament applied to human leg. Doppler ultrasound scanning was used to measure blood flow. Reproduced with permission.^[^ ^95^ ^]^ Copyright 2018, Elsevier Ltd.

Narayana et al.^[^ ^95^ ^]^ reported on the design and optimization of smart stocking structures intended to address limitations associated with conventional PCDs such as cost, noise, bulk, and lack of mobility. Using stress memory polymeric filaments (SMPFs), the design provided static and dynamic (massaging effect) compression, **Figure** **15**. Structural optimization was observed when integrating nylon and SMP filaments (made from polyurethane with a transition temperature of 42.13 °C) into the stocking. A MERZ compression stocking knitting machine was used to fabricate the stocking. Various knitting patterns were tested, varying the ratio of the SMP and nylon fibers spun via melt spinning, and it was noted that the interfacial pressure of the stockings could be controlled by optimizing structural parameters such as float lengths, loop lengths, and loop/stitch density. The stocking was heated up to 50 °C in an experiment using a hair dryer, and the level of massage was controlled by variation of the stocking structure (i.e., the knitting pattern that integrated the SMP yarn with the nylon yarn). The maximum massage effect reached 34 mmHg of compression.

#### 2.3.3 EAPs

EAPs are smart materials that respond to electrical fields by changing shape and dimension. These polymers are easy and cheap to produce and have many advantages including good mechanical flexibility and low density, and so are the subject of significant research interest for use in biomedical applications.^[^ ^37^, ^96^, ^97^, ^98^ ^]^

EAPs can be broadly divided into two categories—namely ionic EAPs which are activated by the transport of ions, and electronic EAPs which are activated by the application of an electric field. Dielectric elastomers (DE) fall into the electronic category, and are among the most commonly investigated EAPs because of their light weight, rapid reaction time, low stiffness, large deformation, and durability. However, DE actuation requires the application of very high electric fields—necessitating the use of several kV—that may pose a risk of electromechanical failure or dielectric breakdown.^[^ ^98^, ^99^, ^100^ ^]^

**Figure 16:**
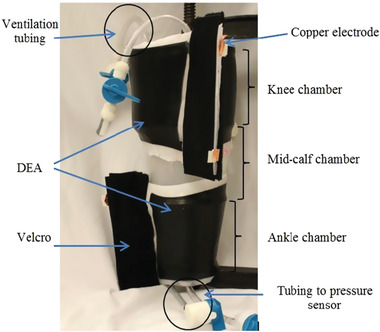
Compression device using a dielectric elastomer actuator. Reproduced with permission.^[^ ^81^ ^]^ Copyright 2014, IOP Publishing.

Pourazadi et al.^[^ ^81^ ^]^ developed a compression bandage prototype based on a DE actuator. They used TC‐5005 silicone elastomer due to its enhanced electromechanical stability, and a mixture of carbon black in ethanol with a concentration of 20 mg mL^−1^ for the electrodes, **Figure** **16**. The sample consisted of a flat dielectric elastomer actuator (DEA) that could be wrapped around the calf using Velcro. A custom‐made voltage box, with a 5 V battery, amplifier, and output range of 2.8–11.3 kV, was used to power the electrodes. The output terminals of the voltage box were connected to copper electrodes in the bandage. The high actuation voltage of the prototype was a major disadvantage, even though the electrodes could be properly insulated and the risk of undesired electrical discharge remained present. A pressure range of 14.4–21.5 mmHg was achieved in the experiments. Advantages of the design included easy don/doff, adaptability to various leg sizes, an ability to tune the compression magnitude of the bandage using pre‐stretch, and a modular structure that allowed a secure fit around different regions of the leg. Each module could be controlled separately, allowing the configuration of massage cycles. Moreover, if a module failed, it could be substituted by a new one without replacing the entire apparatus.

**Figure 17:**
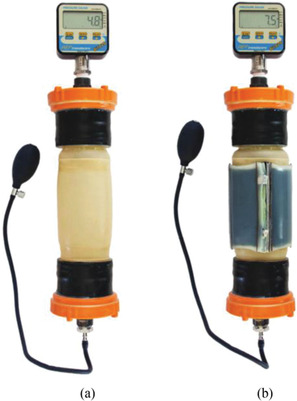
Compression device using electroactive polymer between insulating layers of silicone (dielectric elastomer actuator). Reproduced with permission.^[^ ^101^ ^]^ Copyright 2018, IEEE.

Calabrese et al.^[^ ^101^ ^]^ developed a thin, stretchable electroactive bandage with two pre‐stretched layers and an electroactive acrylic DE film between two insulating layers of silicone elastomer, **Figure** **17**. It used two actuation layers to increase system strength. To optimize actuation in the radial direction, six 2.5 mm diameter sticks made of wood were used during mold casting fabrication of the outer layer. These stiffeners prevented longitudinal elongation of the bandage. Passive layers were used as interfaces to enhance mechanical compliance and electrical safety, and electrical contact to the electrodes was made with metal stripes and carbon black powder. Velcro was glued to the bandage for don/doff. The bandage was designed to be applied with a pre‐stretch to the leg and, when voltage is applied, the bandage released and increased its circumference. The experiments showed that 3 mm was the optimum value of passive layer thickness, and that pressure variation was dependent on the number of electroactive layers, albeit with a saturation trend evident. Applied voltages of up to 8.5 kV resulted in pressure ranges from 6.5 to 7.5 kPa (49 to 56 mmHg). The prototypes were capable of actively varying the applied pressure by up to 10%.

## 3 Discussion

The previous section identified recent research developments in SCT devices that used miniaturized actuators capable of directly or indirectly providing compression to the lower leg. These actuation mechanisms were categorized into three main groups: pneumatic bladders inflated by pumps, straps tightened using electrical motors, and smart materials (SMA, SMP, EAP) integrated into the garments or fabrics. Here, we discuss the primary features of these emerging SCT devices. Section 3.1 compares the relative merits of each actuator category, with a summary presented in **Table** **2**. Section 3.2 assesses the most relevant information relating to the compression systems themselves, with an extensive summary of key characteristics in **Table** **3**.

**Table 2:** Advantages and disadvantages of the actuation mechanisms presented in this state‐of‐the‐art review

Actuation type	Advantages	Disadvantages
Pneumatic^[^ ^55^, ^59^ ^]^	Bladders are flexible, lightweight, and easily conform to the limb shape, which may help with uniform application of pressure; less prone to hysteresis.	Depending on the pump and bladder volume, inflation time to reach desired compression level may be relatively long (≈ 1 min); a pump with small form factor and high flow rate can be costly (≈ 100 USD).^[^ ^104^ ^]^
Motor^[^ ^62^, ^63^ ^]^	Motors can be lightweight (≈ 10 g) and apply relatively high torque, from a wide range (≈ 10 to ≈ 500 N mm^[^ ^105^ ^]^); depending on the tension cable used, the actuation speed can be almost instantaneous.	Securing the motor in a compression garment while high torque is applied can be challenging; may be difficult to generate uniform compression over the limb.
Shape memory alloy^[^ ^53^, ^72^, ^75^, ^76^, ^77^, ^78^, ^79^, ^80^, ^82^ ^]^	SMA's are manufacturable in low profile; can be integrated in a conventional textile to create controlled compression; usually bio‐compatible.^[^ ^70^ ^]^	Difficult to control; low actuation speed; low energy efficiency; prone to hysteresis; requires heat for actuation; slow cooling process.
Shape memory polymer^[^ ^91^, ^92^, ^93^, ^95^ ^]^	Can be integrated in a conventional textile to create controlled compression; pressure distribution can be readjusted by heating the bandage locally or as a whole; generally recyclable; cheaper than SMAs.^[^ ^85^ ^]^	Suffer from stress relaxation over time, resulting in pressure decay; slow activation speed (SMPU cycles take around 10 min^[^ ^92^ ^]^).
Electroactive polymer^[^ ^81^, ^101^ ^]^	Stable, fast response, high stretch ratios, high applicable force, and low cost; withstands high strains, making them suitable as next generation actuators and applicable in many emerging technologies.	High voltages required for actuation (kV level); costly energy sources are needed to deliver the high voltage.

**Table 3:** Key features of the emerging SCT devices identified in this review

Device	Actuation type	Materials and Fabrication	Performance	Observations
Payne et al.^[^ ^55^ ^]^	Pneumatic	Bladder made of thermoplastic elastomer using heat press and Loctite Vinyl Fabrics Adhesive for sealing; used soft force sensor sewn into elastomeric wrap material; Velcro secured the device around the limb; the inner layer of the actuator was sewn with an elasticated warp‐knitted raschel polyamide‐elastane textile; the individual sleeves were sewn together with Kevlar reinforced sailcloth.	Force profiles with amplitudes up to 60 N (0.7 N resolution); pressures up to 10 kPa (75 mmHg); pressure gradients were generated using sequential actuation.	Arrangement of inextensible outer and extensible inner layer directed the force toward the muscles; could mimic manual massage and increase lymph and blood flow.
Hakala et al.^[^ ^59^ ^]^	Pnuematic	Three pairs of bladders were made from polyester fabric coated with polyurethane; bladder plastic inlets were seamed using high frequency welding; normally closed valves controlled airflow; a zipper was located in front of the shin to ease dressing/undressing.	Compression was automated and used a pump and manometer to regulate the pressure inside the bladder; micropump maximum pressure was 400 mmHg; the compression rate could be controlled instantaneously in each sector; reached 50–80 mmHg on a leg model in a few seconds.	A commercial antibacterial understocking was used to protect the skin against friction; prototype weight was 326 g; Bluetooth was used to connect prototype to e‐reader; actuator removable for maintenance if required; a linear relationship between bladder pressure and surface skin pressure was observed; dynamic compression could be provided; warp knit had small holes for ventilation.
Rahimi et al.^[^ ^62^ ^]^	Motor	Used a 12 V DC motor and a lace made from 4‐ply fishing line; motor was fixed on a soccer shin guard using an aluminum l‐bracket; spool and housing were 3D‐printed from ABS plastic and attached to the motor shaft; garment used a non‐stretchable but flexible fabric; Velcro secured the garment.	Pressurized state could be reached in 2 s or less; approximate maximal compression values of 160 and 180 mmHg were observed; accuracy ± 2 mmHg.	Could deliver sustained or intermittent pressure; battery operated and could be used during stasis or ambulation; overall weight was 280 g.
Yang et al.^[^ ^63^ ^]^	Motor	Spun‐silk fabric was used as an inner layer; the outer layer used a single‐layer mesh fabric to provide stiffness in the direction in which a wire was pulled; a motor housing was mounted on a base plate made of thermoplastic polyurethane integrated between the inner and outer layer.	Actuation pressure varied from 19.5 mmHg to 87.3 mmHg.	The sleeve could apply variable or sustained compression, even when the volume of the limb was changed; compact compared to conventional pneumatic actuation devices; difficult to generate a rapid compression because the structure was made of soft material.
Moein et al.^[^ ^80^ ^]^	Shape memory alloy	Actuation was provided by six SMA (NiTi) wires and a 6 mm thick plastic sheet of thermoplastic polycarbonate; the wires were clamped with brass screws, washers, and nuts; a shoelace was used to fasten the compression device.	SMA activation increased pressure up to 9.06 mmHg when applying 219.88 mA (in a 23–31 mmHg total pressure setting).	An analytical model was developed to evaluate the behavior of the compression device.
Holschuh et al.^[^ ^72^ ^]^	Shape memory alloy	SMA NiTi coil actuators were used with a band of 2.54 cm wide breathable fabric and a 3D printed housing to tighten a 200 µm diameter stainless steel cable.	Generated forces up to 24.75 N at 5 V (4.55 W); pressure values could reach up to 6.05 kPa (45 mmHg).	Electrical and thermal insulation for the user was provided; compact and stable voltage‐based actuation; textile and manufacturing challenges remain.
Holschuh et al.^[^ ^75^ ^]^	Shape memory alloy	Cuff‐like compression garment based on NiTi SMA coil actuators and a 3D printed modular cartridge to house the actuators.	27 W (30 V, 0.9 A) was required for 9 s to achieve the pressure targets; maximum counter‐pressure measured was 34.3 kPa (257.3 mmHg).	Capable of delivering dynamic compression; the 3D printed cartridge could generate discontinuities in pressure.
Duvall et al.^[^ ^53^, ^76^ ^]^	Shape memory alloy	A 3D printed structure was used to house the electronics; three main layers included a heat‐resistant textile made from aramid and Lycra, an actuation layer consisting of SMA and a non‐stretch textile, and an outside layer to protect the user from heat.	Average pressure reached a maximum of 37.6 mmHg at 43.8 W.^[^ ^53^ ^]^	Wirelessly controlled using Bluetooth Low Energy; total weight of the vest was ≈0.4–0.6 kg.
Duvall and Pettys‐Baker^[^ ^77^, ^78^ ^]^	Shape memory alloy	Garments were constructed with rip stop nylon and cotton/aramid blends; inner layers were made of Teflon sheets; a fiberglass ribbon tape was sewn vertically with several open channels to keep the actuators in place. SMA was a 0.012″ diameter NiTi alloy wire coiled into a spring with 0.048″ outer diameter.	Maximum average pressure was 17 mmHg.^[^ ^77^ ^]^	Pettys‐Baker et al.^[^ ^78^ ^]^ implemented tension switch; used wireless sensing.
Kennedy et al.^[^ ^82^ ^]^	Shape memory alloy	SMAs consisting of Ni‐Ti‐Cu wires were integrated inside a woven‐textile and assembled in a commercial stocking.	The actuator generated a pressure surplus between 0.3 mmHg and 1.8 mmHg over the compression garment starting pressure.	Ni‐Ti‐Cu wires were intended to facilitate good recovery and control of other features such as garment elastic displacement, safety, and temperature; the design insulated the user from heat; heating time and cooling times were 5 s and 15 s, respectively.
Granberry et al.^[^ ^79^ ^]^	Shape memory alloy	Based on SMA Flexinol wire and Kevlar aramid fiber yarn.	Activation temperature was 70 °C; 120 W total power consumption; no pressure data reported.	This device was an active knit compression stocking.
Ahmad et al.^[^ ^79^ ^]^	Shape memory polymer	Polyurethane‐based shape memory polymer (SMPU); strips were attached to a fabric which was part of the bandage underlay padding using Araldite Rapid adhesive.	Transition temperatures of the SMPU were ≈ 50 °C. Applied pressure range was 10 mmHg to 45 mmHg.	Good biocompatibility; SMPU could be reused or recycled many times; resilient against body fluids; stress relaxation from cycling was evident.
Kumar et al.^[^ ^92^ ^]^	Shape memory polymer	Polyurethane film SMP; polyester was used as base fabric and copper wire as heat source; fabric layers were stitched together to secure the position of the heating layer; Velcro was used for fastening around the limb.	Actuation temperature 30 to 60 °C; up to 40 mmHg pressure; slow actuation speed, which could take 10 min to complete a massage cycle.	The device could provide dynamic compression if an alternating temperature stimulation profile was provided.
Kumar et al.^[^ ^92^, ^93^ ^]^	Shape memory polymer	Blend yarn of polyurethane‐based SMP and nylon knitted using a V‐bed double jersey flat weft machine.	Activation range of 30 to 50 °C; extra pressure up to 16.2 mmHg was generated (46.2 mmHg maximum total pressure).	Used the stress memory phenomenon of the memory polymer.
Narayana et al.^[^ ^95^ ^]^	Shape memory polymer	Integrated polyurethane and nylon into stocking filaments; a MERZ compression stocking knitting machine was used to fabricate the stocking.	When actuated, the extra pressure provided by the stocking reached up to 34 mmHg.	The device could deliver both dynamic and static pressure.
Purazadi et al.^[^ ^81^ ^]^	Electroactive polymer	Dielectric elastomer actuator (DEA) consisting of TC‐5005 silicone and electrodes made from a carbon black/ethanol mixture; Velcro was used to secure garment.	Voltage supplied for actuation ranged from 2.8 to 11.3 kV; a pressure of 14.4–21.5 mmHg was recorded.	Silicone elastomer was used due to its enhanced electromechanical stability; the high actuation voltage is a notable disadvantage; each module could be controlled separately, end therefore enables massage cycles; if a module failed, it could be substituted without replacing the entire apparatus.
Calabrese et al.^[^ ^101^ ^]^	Electroactive polymer	Fabricated using electroactive acrylic elastomer and insulating layers of silicone; six wooden sticks were used in the construction of the outer passive layer; the electrodes were made of carbon black powder and electrical contact was made with metal stripes integrated within the structure; two active layers were stacked together to increase the strength of the system.	Applied voltage ranged from 0–8 kV; pressure was 18.8–56.2 mmHg.	The passive outside layers provided electrical insulation; wooden stiffening sticks maximized actuation in radial direction; the device was applied with a pre‐stretch that released when voltage was applied, which reduced the applied pressure.

### 3.1 Actuator Comparison

As mentioned in the introduction, some of the main design requirements for SCT devices include 1) the ability to provide compression within the desired pressure range; 2) low energy consumption; 3) rapid pressure adjustment; 4) wireless connectivity; 5) continuous monitoring of pressure levels; 6) a lightweight and comfortable form factor. In the context of these requirements, the primary advantages and disadvantages of each actuator category are discussed in this section and summarized in Table 2.

The maximum applied pressure that could be achieved was strongly dependent on the type of actuation used. The pneumatic bladder solutions reached up to 80 mmHg, and motor‐driven devices achieved up to 180 mmHg. Studies on smart material‐based compression devices reported a significant range of maximum pressure, from 1.8 up to 257 mmHg. The application of specific pressures using compression garments and smart materials is a challenging prospect due to the material transformation complexities, as well as risks associated with high voltages or temperatures outlined earlier. Motors seem to have good potential for use in higher pressure applications; however, integrating a lacing system in the garment can also be challenging. The successful application of controllable compression within a particular pressure range, regardless of actuation type, is also heavily dependent on how the actuator is integrated with the garment.

Operational lifetime will obviously depend on the power consumption of the actuators. Motors and pumps with small form factors have power consumption ranging from ≈50 mW to 10 W and can be used to apply higher pressures than those achievable with most smart material solutions. The devices with smart materials presented in this paper have power consumption ranging from 4.55 W to 120 W, necessitating the use of relatively large energy sources. Therefore, motors and pumps are more energy efficient and can enable more autonomy in a mobile device for a given battery size.

Regarding actuation speed, motor‐based mechanisms are most responsive, typically reaching maximum pressure values in as little as 2 s. The actuation of pneumatic devices depends on the bladder design and air flow rate delivered by the micropump. Smart materials have a slow actuation relative to the maximum pressure that they can apply, especially SMPs, which can have an actuation cycle of up to 10 min.

The development of electronic subsystems for wireless communication and device control is independent of the actuation mechanism and should be based on the specific use case and user needs for that device at hand. Continuous monitoring of the applied pressure is a key requirement for SCT systems, and this could also be made independent of actuation principle, that is, it is possible to integrate various sensors for compression measurement—most notably ultrathin pressure sensors^[^ ^102^, ^103^ ^]^—with each system, and these are outlined in more detail in Section 3.2. However, several approaches also have inherent characteristics (e.g., air pressure in the case of pneumatics; strain in the case of smart materials) that could also be measured and be used to indirectly infer surface pressure. This would eliminate the cost and complexity associated with incorporating an additional sensor specifically for surface pressure measurement. However, the variation in limb sizes/shapes and don/doff procedures means that achieving a reliable correlation between this characteristic and applied surface pressure could be challenging.

As SCT devices are intended to be worn for significant periods of time and in ambulatory settings, weight and portability are obviously key concerns. It is possible to find pumps and minimotors weighting as little as 5 g,^[^ ^104^ ^]^ which is insignificant compared to the weight of the complete device. However, bladders and/or lacing mechanisms must also accompany this core component. Smart materials, if integrated within a garment to compose a stocking, add very little extra weight and form to the garment. In this case, the overall weight will be most likely limited by the power supply and electronic hardware for the actuation of the smart materials.

### 3.2 Device Comparison

Seventeen different SCT devices, described in twenty research articles, were identified during the course of this review and are summarized in Table 3. Reflecting the current potential of SCT and interest in smart materials, the number of devices found in each different actuation category were: pneumatic‐2; motor‐2; SMA‐7; SMP‐4; and EAP‐2.

Depending on the actuator selected, the garment may need specific additional features to house the mechanism and to transduce the input energy into surface pressure on the limb. For example, when a pump inflates a bladder, this pneumatic system must be integrated in the device using tubes and/or valves.^[^ ^55^, ^59^ ^]^ When a motor is used with a lacing system, structures are required to ensure the uniform constriction of the limb, and this may be achieved in several ways—Rahimi et al.,^[^ ^62^ ^]^ for instance, installed the motor on a soccer shin guard. Alternatively, Yang et al.^[^ ^63^ ^]^ mounted the motor on a base plate, and pulled on two bars that in turn were attached to the garment itself.

As a result, it is also clear that engineering methodologies such as Design for Manufacture and Assembly (DFMA)^[^ ^106^ ^]^ must be taken into account at a very early stage in the development process. Emerging SCT devices represent heterogeneous assemblies of fabrics, sensors, actuators, electrical circuitry, and power sources, and therefore the design requirements are much more complex than those associated with conventional, textile‐only compression garments. The use of such dissimilar textiles, actuators, and components requires additional considerations not directly related to the provision of compression, including, for example, the need for specific materials to provide thermal or electrical insulation,^[^ ^76^, ^78^ ^]^ the safe and wearable provision of high electrical currents or voltages,^[^ ^53^, ^78^, ^81^, ^101^ ^]^ solution of issues surrounding additional pressures generated by the components themselves,^[^ ^53^ ^]^ or breathability limitations due to the multiple layers necessary for SCT construction.^[^ ^92^ ^]^ In addition, the addition of electronic and/or materials hardware must not detract from the primary objective—to apply compression at clinically relevant levels, in a form factor that is acceptable to the patient and that offers worthwhile advantages over conventional garments.

Systems based on smart materials may be subdivided into those that are not integrated with the textile itself, and those that are. The former is the most commonly used option among the articles reviewed for this paper. In that case, that is, where the actuator is not integrated with the garment, the pressure is instead applied by pulling the garment's fabric as demonstrated using SMA^[^ ^53^, ^72^, ^75^, ^76^, ^77^, ^78^, ^80^, ^82^ ^]^ or by constricting the actuator around the limb using either SMP or EAP materials.^[^ ^81^, ^91^, ^92^, ^101^ ^]^ For example, Kennedy et al.^[^ ^82^ ^]^ integrated a SMA wire in a compression stocking, where the actuator pulled on the fabric to provide the compression of the limb. Other works also used SMA materials^[^ ^72^, ^75^, ^77^, ^80^ ^]^ in the form of wires or coil actuators to pull on the fabric and compress the limb. The same principle was demonstrated in a torso vest,^[^ ^53^, ^77^ ^]^ but this same technology could also be implemented in a leg‐worn garment to treat VLUs. Some devices used SMP and EAP actuators that also were not integrated into the fabric, including, for example, the garments developed by Ahamad et al.,^[^ ^91^ ^]^ which attached SMP strips to the garment. Kumar et al.^[^ ^92^ ^]^ used a film of SMP made of polyurethane. All devices using EAP as actuators did not integrate the EAP with the textile, but instead used plates made of EAP to apply the compression.^[^ ^81^, ^101^ ^]^

Four works described devices where a smart material yarn was knit as an integral part of the garment textile. Granberry et al.^[^ ^79^ ^]^ mixed Kevlar aramid fiber yarn with SMA flexinol wire in a stocking with three sections, each using a different concentration of the fibers in order to generate a tapered compression profile. Other works^[^ ^92^, ^93^, ^95^ ^]^ proposed the use of SMP and nylon in the stocking filaments.

An important component of a device that allows it to function as a smart device is a sensor,^[^ ^40^, ^107^ ^]^ which at the very least provides direct or indirect pressure monitoring and could ultimately allow closed‐loop control of the actuation mechanism. Most works presented here used external sensors for surface pressure monitoring during the development phase, and these were not usually integrated into the whole garment. However, even if the sensors were integrated in the garment, just a few used a feedback loop to regulate the pressure at present,^[^ ^55^, ^59^, ^63^ ^]^ and implementation of this ability to autonomously control applied pressure will be key to the successful deployment of SCT technologies in the future. A sensor commonly used to evaluate under‐bandage pressure is the Kikuhime device,^[^ ^108^ ^]^ which was applied in several works presented here.^[^ ^91^, ^92^, ^93^, ^95^ ^]^ Hakala et al. used the PicoPress sensor for skin pressure evaluation.^[^ ^59^, ^109^ ^]^ Other sensors used for evaluation of the technologies reviewed here were force sensors from Novel,^[^ ^72^, ^75^ ^]^ Interlink Electronics,^[^ ^59^, ^62^ ^]^ and Tekscan.^[^ ^53^, ^55^, ^59^, ^77^ ^]^ While most works used commercial sensors, it is interesting to note also that Payne et al.^[^ ^55^ ^]^ used innovative silicone‐textile capacitive sensors in their garment. It should be highlighted also that scope exists to create multifunctional wound treatment systems by merging these smart compression dressings with other sensor‐related aspects of new woundcare technologies, most notably those that seek to monitor the status of the dressing and of the wound itself using sensors for pH, temperature, oxygenation, moisture, and various biomarkers for infection control.^[^ ^46^, ^107^, ^110^ ^]^

A wide variety of materials was used in the construction of the various prototypes, including silk, cotton, fiberglass, Lycra, Kevlar, aramid, nylon, Teflon, polyester, Velcro, polyurethane, and 3D printed components. The highest reported compression of over 257 mmHg was reached by Holschuh et al.^[^ ^75^ ^]^ using a SMA‐based approach, and the lowest (1.8 mmHg) by Kennedy et al.,^[^ ^82^ ^]^ also using SMA. This shows the wide range of pressures that SMA‐based technologies can provide. In the case of fully integrated smart material/textile devices, the highest pressure reported was 46.2 mmHg;^[^ ^92^, ^93^ ^]^ although in this case a significant baseline pressure was exerted solely due to the donning procedure and SMA actuation was then used to controllably generate additional pressure.

Table 3 summarizes the key characteristics of the technologies presented earlier in this paper. It includes brief details of actuation type, materials and fabrication, performance, and some observations of each device.

## 4 Conclusion

This paper highlights recent research effort in SCT devices, which we define as those systems that use miniaturized and lightweight actuators and electronics to control the pressure applied to a limb. A total of seventeen different SCT devices have been identified, which were categorized by operating principle into one of three types: pneumatic compression, motor‐driven mechanisms, and smart materials (including SMAs, SMPs, and EAPs). The design features, performance, and key characteristics of each device have been discussed.

All of the actuation technologies presented here have the potential for efficient and effective VLU treatment. The field is still in its relative infancy and further technology refinements are required in order to create mass manufacturable compression dressing systems that meet medical, ergonomic, and economic standards. Of the 20 research articles that were identified during this review, a total of 16 used a smart material for the pressure application, illustrating the potential of this technology for use in compression garments. Smart materials have important advantages, including flexibility and ease of integration, that are attractive for smart dressings. Furthermore, smart materials could be integrated in the garment as blend yarn, adding virtually no additional structure to the garment. However, these materials require complex control mechanisms, which pose safety considerations and require the provision of additional hardware to generate high voltages and/or temperatures. The use of pneumatic and motor‐driven combinations are typically a little more bulky and complex to integrate with the dressing, but the actuation sources themselves are relatively well developed and consume lower levels of energy compared to the smart materials. These tradeoffs between complexity, accuracy, energy consumption, cost, size, and weight must be carefully considered, and it is possible that a hybrid combination of the actuation/sensing mechanisms presented here could be a solution for future devices.

The research reviewed in this paper has shown that the use of miniaturized actuators has immense potential for the development of smart compression dressings. Continued progress in this area will ultimately lead to enhanced compliance, patient comfort, mobility, and better treatment outcomes, and it is clear that a high social and economic impact would result from the successful development of this emerging technology.

## Conflict of Interest

The authors declare no conflict of interest.
